# The Interactions between Innate Immunity and Microbiota in Gastrointestinal Diseases

**DOI:** 10.1155/2015/898297

**Published:** 2015-05-20

**Authors:** Danilo Pagliari, Ciriaco A. Piccirillo, Anis Larbi, Rossella Cianci

**Affiliations:** ^1^Department of Medical Sciences, Catholic University of Rome, Largo A. Gemelli 8, 00168 Rome, Italy; ^2^Department of Microbiology & Immunology and Medicine, Montreal General Hospital, McGill University and Research Institute of McGill University Health Centre (MUHC), Montreal, QC, Canada; ^3^Singapore Immunology Network (SIgN), Singapore

The gut microbiota refers to the collection of microbial populations that reside in the gastrointestinal tract. It is characterized by an interplay between different cell types and their defense systems, food particles, molecules derived from digestion, and the vast array of residing microbial species with their secretory products. These microorganisms present in the gut lumen, which can be classified as probiotics, commensals, or pathogens, form the microbiota, which exerts several physiological functions, that is, the absorption and digestion processes, tolerance to non-self-food antigens, and defense from pathogens. The human microbiota can weigh up to 2 kg in total and contains tens of trillions of microorganisms, a number 10 to 20 times greater than the total number of cells in the human body, and includes at least 1000 different bacterial species [1]. This rich gut microbial community has coevolved in a symbiotic relationship with the human intestinal mucosa in such a way that the indigenous microbiota is essential for gut homeostasis. This ecosystem acts as a functional unit; thus, microbiota is considered a “superorganism” and is an integral part of the gastrointestinal tract [2].

The human intestine has the dual function of nutrient absorption and protection against intestinal pathogens. These two tasks have conflicting demands in the body. Optimal absorption requires a large surface area and selective permeability to nutrients through a thin epithelial layer. However, a large surface area increases exposure to luminal bacteria, and the thinness of epithelium increases the possibility of bacterial invasion. The intestinal immune system acts in order to avoid bacterial invasion of the epithelial layer. At the same time, it must not initiate inappropriate immune responses towards the commensal bacteria that reside in the intestinal lumen, as these responses may result in inflammatory diseases, such as Inflammatory Bowel Diseases (IBD), Celiac Disease (CD), and Diverticular Disease. Accordingly, it is important for the intestinal immune system to maintain an appropriate balance of pro- and anti-inflammatory mechanisms to ensure tolerance to commensal and dietary antigens and intestinal homeostasis [3].

The relationship between gut flora and humans is not merely commensal but rather a symbiotic one. This relationship is constantly challenged by many factors, such as the rapid turnover of the gastrointestinal epithelium and mucus, the exposure to the peristaltic activity, the presence of molecules resulting from digestion, the pancreatic and biliary secretions, the host defense, the presence of drugs or medications, the changes in pH and redox potential, and the exposure to transient organisms from the oral cavity and esophagus. Alternatively, numerous beneficial functions are ascribed to the microbiota in the human gut such as fermenting unused energy substrates, educating the immune system, preventing growth of harmful, pathogenic bacteria, regulating gut development, producing vitamins for the host, such as biotin and vitamin K, and producing hormones to direct the host to store fats [4]. Furthermore, the gut microbial community is akin to a safeguard of our health because the microbiota competes for space and nutrients with potential pathogens and induces the secretion of antimicrobial peptides through interaction with intestinal epithelial cells [1]. The gut microbiota can also stimulate the differentiation and proliferation of epithelial cells, which regulate intestinal homeostasis [5].

According to the beneficial functions of gut microbiota mentioned above, it is clear that several bacteria that form microbiota confer a health benefit on the host. These bacteria have been mentioned as “probiotics.” Thus, in recent years, experimental and clinical researchers have identified probiotics as a potential therapeutic target for several gastrointestinal diseases, as reported in the review by G. Giorgetti et al. Quali-quantitative changes in bacterial strains suggest that gut microbiota may be a therapeutic target based on probiotics and on probiotic-driven metabolic products, called “postbiotics.” New therapeutic approaches based on probiotics are now available, and further treatments based on postbiotics will come in the future.

A principal function of the microbiota is to protect the intestine against colonization by exogenous pathogens and potentially harmful indigenous microorganisms. The specific mechanisms by which commensal bacteria achieve this function remain poorly understood, but there is evidence to indicate that both direct and indirect mechanisms might be involved. These mechanisms include the direct competition for limited nutrients, the promotion of mucosal barrier function, the modulation of innate immunity to pathogens and infections, the promotion of adaptive immunity, and even the protective role of the commensal microbiota against systemic infection [1]. However, in certain conditions, some species of bacteria are thought to cause disease by producing infection or increasing the risk of cancer in the host. In fact, particular bacterial populations that are typically found in very low abundance can acquire pathogenic properties. These conditions include inherent immune defects as well as changes in diet and/or acute inflammation and can result in the disruption of the normal balanced state of the gut microbiota, a condition referred to as dysbiosis [6]. Dysbiosis involves the abnormal accumulation or increased virulence of certain commensal populations of bacteria, thereby transforming former symbionts into “pathobionts.” Pathobionts are typically colitogenic in that they can trigger intestinal inflammation [1]. Hence, the breakdown of the normal microbial community contributes to increase of the risk of pathogen infection, the overgrowth of harmful pathobionts, and inflammatory disease. Thus, understanding the interaction of the microbiota with pathogens and the host might provide new insights into the pathogenesis of disease, as well as novel avenues for preventing and treating intestinal and systemic disorders [1].

A prototype to understand the breakdown of the normal gut microbial community leading to dysbiosis is* Clostridium difficile* infection (CDI). In this special issue, S. Bibbò et al. describe the epidemiological and clinical characteristics of CDI and discuss how the intestinal microbiota modifications and the modulation of innate immune response can promote and exacerbate CDI. In this infection, the alteration of intestinal homeostasis promotes the development of an ecological niche that allows the growth of* Clostridium difficile.* Moreover, intestinal dysbiosis can promote a proinflammatory environment, whereas* Clostridium difficile* itself modulates the innate immunity through both toxin-dependent and toxin-independent mechanisms.

CDI causes intestinal inflammation leading to a subsequent reduction of intestinal microbiota diversity. The proportion between* Clostridium difficile* and the other gut bacteria has been related to the severity of inflammation. The work of C. Vincent et al. demonstrates how fecal excretion of abundant quantities of human DNA is associated with intestinal inflammation and with incipient CDI; thus, fecal excretion of host DNA should be investigated as a potential clinical marker of intestinal inflammation and CDI risk.

The specific composition of the microbiota is influenced by immune cells as well as external environmental factors, especially the use of antibiotics and diet [7]. For the preservation of gastrointestinal homeostasis, the intestine maintains control over the microflora, discriminating between pathogenetic and nonpathogenetic organisms by means of local innate immunity [1].

Initiation of innate immune responses in the intestine is triggered by pathogen-recognition receptors (PRRs) which serve as sensors of pathogen-associated molecular patterns (PAMPs) from the intestinal lumen. The most studied PRRs are the Toll-like receptors (TLRs) [8]. In this special issue, two reviews illustrate the major role of TLRs in the interaction between microbiota and immune system. In particular, M. Valentini et al. focus the attention on the possibility that microbiota acts through TLRs expressed by adaptive T cells providing regulatory signals. S. Frosali et al. discuss the role of TLRs polymorphism in human gastrointestinal pathology and the role of TLRs and their interactions with human microbiota in the pathogenesis of Inflammatory Bowel Diseases.

TLRs may be involved in the pathogenesis and clinical course of the diverticular disease. In the paper by R. Cianci et al., it has been demonstrated that variations before and after the antibiotic therapy with Rifaximin exist. This suggests that TLRs are modified by the presence of pathogenic flora in UDD. The role of pathogenic flora is supported by the finding that Rifaximin acts in the gut mucosa homeostasis by limiting the activation of TLRs. Furthermore, Rifaximin may also have a luminal anti-inflammatory function modulating the adaptive immune response in an inhibitory sense. Rifaximin keeps under control TLRs expression in peripheral blood suggesting that, in addition to its activity in gut mucosa, it may also have a systemic action on immune system.

The intestinal barrier regulates intestinal homeostasis throughout innate and adaptive immune responses. Unfortunately, in some cases, the innate immune system's attempt to protect the host fails and chronic inflammation and intestinal autoimmunity occur, such as in the case of IBD and Celiac Disease (CD). In this special issue, in the paper of D. Pagliari et al. it is clarified how CD may be considered as a model to explain the pathogenesis of several other autoimmune diseases. In fact, during this disease, the steady state condition homeostasis due to the complete balance between pro- and anti-inflammatory factors is altered. The breakdown of the interaction among microbiota, innate immunity, and genetic and dietary factors leads to the disruption of homeostasis leading to inflammation and tissue damage. Thus, focusing the attention on this interaction and its breakdown may allow a better understanding of the CD pathogenesis and finally get novel translational avenues for preventing and treating this widespread disease.

The contributions of the gut microbiota to the development of the immune system have been extensively characterized. There is a coordinated cross talk between the gut microbiota and the immune system allowing the host to tolerate the large amount of antigens present in the gut. Hence, recent data have demonstrated that microbiota plays a pivotal role in orchestrating the immune response in gut mucosa and in regulating the balance between pro- and anti-inflammation factors. Gut flora has a continuous and dynamic effect on the host's gut and systemic immune systems. Bacteria are key in promoting the early development of the gut's mucosal immune system. The immune system recognizes and fights harmful bacteria, but, throughout the mechanism of tolerance induction within the first days of life, permits the colonization and growth of helpful species of bacteria. On the other hand, the immune-gastrointestinal flora interface plays a pivotal role in maintaining gut homeostasis with resident microbial communities, thus ensuring that the symbiotic nature of the host-microbial relationship is maintained [2].

According to what has been described so far, the interaction between human gut microbiota and intestinal immune system can be considered as the pivotal axis that contributes to the maintenance of intestinal homeostasis and allows proper defense to infections. As the intestinal immune system is influenced by many factors, including dietary components and commensal bacteria, strategies that restore a healthy gut microbial community by affecting the microbial composition are being developed as new therapeutic approaches to treat several gastrointestinal diseases.

We hope that this special issue will contribute to a better understanding of the strong interactions between innate immunity and microbiota in developing gastrointestinal diseases. These new acquisitions will be fundamental to finding new therapeutical strategies.

*Danilo Pagliari* *Danilo Pagliari* *Ciriaco A. Piccirillo* *Ciriaco A. Piccirillo* *Anis Larbi* *Anis Larbi* *Rossella Cianci* *Rossella Cianci*
